# Rhizobial Inoculation Increases Soil Microbial Functioning and Gum Arabic Production of 13-Year-Old *Senegalia senegal* (L.) Britton, Trees in the North Part of Senegal

**DOI:** 10.3389/fpls.2016.01355

**Published:** 2016-09-07

**Authors:** Dioumacor Fall, Niokhor Bakhoum, Saïdou Nourou Sall, Alzouma Mayaki Zoubeirou, Samba N. Sylla, Diegane Diouf

**Affiliations:** ^1^Centre National de Recherches Forestières, Institut Sénégalais de Recherches AgricolesDakar, Senegal; ^2^Laboratoire Commun de Microbiologie, Institut de Recherche pour le Développement/Institut Sénégalais de Recherches Agricoles/Université Cheikh Anta DiopDakar, Senegal; ^3^Laboratoire Mixte International Adaptation des Plantes et Microorganismes Associés aux Stress EnvironnementauxDakar, Senegal; ^4^Unité de Formation et Recherche des Sciences Agronomiques de l’Aquaculture et des Technologies Alimentaires (UFR S2ATA) – Université Gaston BergerSaint-Louis, Senegal; ^5^Département de Biologie Végétale, Université Abdou MoumouniNiamey, Niger; ^6^Département de Biologie Végétale, Université Cheikh Anta DiopDakar, Senegal

**Keywords:** rhizobia, plant productivity, gum arabic, soil fertility, mineralization, arid regions

## Abstract

Rhizobial inoculation has been widely used in controlled conditions as a substitute for chemical fertilizers to increase plants growth and productivity. However, very little is known about such effects on mature trees in natural habitats. In this study, we investigated the effect of rhizobial inoculation on soil total microbial biomass, mineral nitrogen content, potential CO_2_ respiration, fluorescein diacetate (FDA), acid phosphatase activities, and gum arabic production by 13-year-old *Senegalia senegal* (synonym: *Acacia senegal*) under natural conditions in the north part of Senegal during two consecutive years. Rhizobial inoculation was performed at the beginning of the rainy season (July) for both years with a cocktail of four strains (CIRADF 300, CIRADF 301, CIRADF 302, and CIRADF 303). Rhizospheric soils were collected in both dry and rainy seasons to a depth of 0–25 cm under uninoculated and inoculated trees. Trees were tapped in November (beginning of dry season) using traditional tools. Gum arabic was harvested every 15 days from December to March. The results obtained from both years demonstrated that rhizobial inoculation increased significantly the percentage of trees producing gum arabic, gum arabic production per tree, soil microbial biomass, FDA, and acid phosphatase activities. However, there was no significant effect on C mineralization and mineral nitrogen (N) content. Gum arabic production was positively correlated to rainfall, soil microbial biomass, and mineral nitrogen content. Our results showed a positive effect of rhizobial inoculation on soil microbial functioning and gum arabic production by mature *S. senegal* trees. These important findings deserve to be conducted in several contrasting sites in order to improve gum arabic production and contribute to increase rural population incomes.

## Introduction

Severe environmental conditions in arid and semi-arid areas worsen food insecurity in Sub-Saharan African countries. A potential solution to the declining of soil fertility caused by shortened fallow period is using nitrogen fixing trees in agroforestry systems (Dommergues, 1995). While nitrogen fixing trees are promising substitutes for chemical fertilizers for resource-poor farmers in the arid regions of Africa (Dakora and Keya, 1997) more efficient inoculation techniques using effective symbionts are needed to ensure optimal exploitation of their economic and agricultural potential (Dommergues, 1995). Sarr et al. (2005a) showed that using alginate beads containing rhizobial cells improved efficiently growth performance in *Senegalia senegal* seedlings under nursery conditions. Likewise, similar results obtained with the same technique were reported under irrigation (Sarr et al., 2005b; Fall et al., 2011; Bakhoum et al., 2016). In arid and semi-arid areas, species with multi-purpose are more suitable for reforestation. Therefore, *S. senegal* (L.) Willd. has received more attention, due to its inherent ability to fix atmospheric nitrogen (N_2_) which eventually returns to the soil through N-rich litter-fall, root and nodule turnover. *Senegalia* and *Vachellia* species are widely distributed in Sub-Saharan Africa region where they are used in reforestation processes (Midgley and Bond, 2001). *S. senegal* has considerable potential in agroforestry systems, fuelwood production, forage, and medicinal products. The species contributes to soil conservation and enhancement of soil fertility in agroforestry systems, and is used by farmers in the arid and semi-arid zones of Africa for gum arabic production (Von Maydell, 1983). Gum arabic is a highly valued natural resource for rural populations in the Sahel, particularly in dry land populations where it represents an important source of income (Muller and Okoro, 2004). In such areas, sustainable management is required to enhance local production. Gum arabic exudation is a complex and poorly understood process which is still not completely known today. Natural exudation is caused by traumatisms such as wind, dryness, human activity, and animals (Sall, 1997). Gum arabic exudation is usually attributed to tree water stress during the dry season (Sall, 1997). Gum arabic yield can also be influenced by tapping date and environmental factors such as temperature and rainfall (Ballal et al., 2005). In addition to these abiotic factors, the amount of gum arabic produced by tree may be affected by mineral nutrition and/or physiological properties of the tree, which depend on soil fertility and/or the tree vigor. Indeed, in Senegal, gum arabic producers have observed that the most vigorous trees with very green leaves in rainy season produce most of gum arabic during the following harvest. Many studies showed that microbial inoculation improves significantly growth, physiological and biochemical parameters of seedlings in greenhouse (Galiana et al., 1990; Dommergues, 1995; Diouf et al., 2003; Fall et al., 2011; Bakhoum et al., 2016). However, very few studies were conducted under natural conditions on the effect of rhizobial inoculation on growth and productivity of mature trees and soil microbial functioning (Faye et al., 2006; Assigbetsé et al., 2012). While in such conditions, biotic and abiotic factors such as rainfall can affect negatively N_2_-fixing capacity, legumes productivity, and soil fertility. This study was investigated to determine under natural conditions and during two consecutive years, the effect of rhizobial inoculation on gum arabic production of 13-year-old *S. senegal* trees and soil microbial functioning. Soil total microbial biomass, inorganic carbon and nitrogen content, fluorescein diacetate (FDA), and acid phosphatase activities were the measured parameters for soil microbial functioning. The relationship between these parameters and gum arabic production was also investigated.

## Materials and Methods

### Site Description and Experimental Design

The experiment was conducted at Kamb (15°32′N, 15°26′W), an arid savanna located at 300 km in the north of Dakar (Senegal) where total annual rainfall varies between 400 and 500 mm (Fall et al., 2012). In this part of Sahel, temperatures vary between 25 and 39°C with an average of approximately 29°C. The experimental site was an *S. senegal* plantation of 4 ha, and trees planted on a grid of 5 m × 5 m were never tapped. At the beginning of the experiment (middle of dry season, April), the plantation was divided into two homogenous blocks each of which containing approximately 106 trees. These two blocks were divided into two plots (1 ha each), inoculated trees and control or uninoculated trees. There were then four plots (two uninoculated and two inoculated) and results presented here are the mean values of same treatments (uninoculated vs. inoculated). Collar diameter and height of trees were similar in all plots (**Table 1**). Annual rainfall was recorded during the 2 years (**Table 2**) in order to estimate the correlation between annual rainfall and gum arabic yield.

**Table 1:** Collar diameter and height of 13-year-old *S. senegal* trees measured prior the experiment.

Treatments	Tree height (m)	Collar diameter (cm)
Uninoculated trees	3.79 ± 0.7a	52.01 ± 10.45a
Trees to inoculate	3.80 ± 0.9a	51.28 ± 9.85a

*Values within a column sharing same letter are not significantly different at *P* < 0.05 (Student–Newman–Keuls test).*

*Each value represents the mean of 106 repetitions (53 per block × 2 blocks).*

**Table 2:** Rainfall data (mm) recorded in the experimental site during the 2 years.

	First year					Second year				
	June	July	August	September	October	June	July	August	September	October
NRD	2	7	8	8	1	1	3	8	7	1
MR	69.4	139.1	128.8	151.2	7.1	9.2	62.6	74.8	53	13.5
AR	495.6					213.1				

*NRD, number of raining days; MR, monthly rainfall (mm); AR, annual rainfall (mm).*

### Soils Sampling

Soils samples were collected in dry (April) and rainy (August, after inoculation) seasons during the two consecutive experimental years as described by Fall et al. (2012). For each sampling period, soils were collected in each plot at the foot of six mature *S. senegal* trees that were separated by a distance of at least 10–15 m. For each tree, soil samples were collected from four directions (East, West, North, and South) at a depth of 0–25 cm and pooled to get a homogenous soil sample around the tree. Then for each treatment (inoculated and uninoculated), we had 12 soil samples (six in each block) analyzed separately, and each value presented in tables was the mean of 12 repetitions. Soils chemical characteristics are presented in **Table 3**.

**Table 3:** Chemical characteristics of soils collected prior the experiment at 0–20 cm layer under uninoculated trees and trees to inoculate.

Treatments	pH_H2O_	NH_4_^+^ + NO_3_^-^	Org. C	Sol. P
Uninoculated trees	5.3 ± 0.34a	09.1 ± 2.45a	0.23 ± 0.02a	29.8 ± 3.31a
Trees to inoculate	5.8 ± 0.20a	12.8 ± 1.14a	0.25 ± 0.01a	27.3 ± 2.04a

*Values within a column sharing same letter are not significantly different at *P* < 0.05 (Student–Newman–Keuls test).*

*NH_4_^+^ + NO_3_^-^ (mg/kg of soil); Org. C, organic carbon (%); Sol. P, soluble phosphorus (mg/kg of soil).*

### Inoculation of *S. senegal* Trees

*Senegalia senegal* trees were inoculated at the beginning of rainy season (July) for each experimental year with alginate beads containing rhizobial cells prepared according to Diem et al. (1989). The four rhizobial strains used (CIRADF 300, CIRADF 301, CIRADF 302, and CIRADF 303) belong to *Ensifer* genus (Sarr et al., 2005b; Sarr and Lesueur, 2007). Five grams of dried beads were dissolved in 1 l of a phosphate buffer (23 g K_2_HPO_4_ and 14.6 g of KH_2_PO_4_), to produce 1 l of liquid inoculum, which was then stirred overnight before proceeding to inoculation. Inoculation was done by pouring slowly 1 l (10^7^ bacterial cells/ml) of this solution around the trunk of each tree. Uninoculated trees had received the same amount of phosphate buffer without rhizobia.

### Trees Tapping and Gum Arabic Harvests

Trees were tapped at the beginning of dry season (November) using traditional tools and at intensity of eight wounds per tree (on the same size of branches in order to ensure treatments comparison). The gum arabic production started 2 weeks after tapping. Gum arabic was harvested every 15 days from December to March for each harvested season. Gum arabic harvested was dried and weighed. Results were expressed in amount of gum arabic produced per tree every 15 days, percentages of trees producing gum arabic and amount of gum arabic produced per tree at the end of harvest season.

### Determination of Soil Total Microbial Biomass

Soil microbial biomass was determined using the fumigation-extraction method (Amato and Ladd, 1988). Fumigated and unfumigated soil samples were suspended in KCl 1 M solution, shaken at 25°C for 2 h and then filtered. Ninhydrin-reactive N content was determined by flow injection analysis (Evolution II, Alliance-Instruments, France). Microbial biomass C was estimated from the gain in ninhydrin-reactive N after a 10-day fumigation period, multiplied by 21 (Amato and Ladd, 1988). Microbial biomass was expressed in μg C g^-1^ of dry soil.

### Assessment of Inorganic N (NH_4_^+^ + NO_3_^-^) Content and Microbial CO_2_ Respiration

Soil inorganic N (NH_4_^+^ + NO_3_^-^) content in KCl extracts (KCl 2 M) was determined by flow injection analysis according to Bremner (1965) method. The results were expressed in μg N (NH_4_^+^ + NO_3_^-^) g^-1^ of dry soil.

For microbial CO_2_ respiration, 10 g of each soil sample (under uninoculated and inoculated *A. senegal* trees) were weighed. Soils moisture content was adjusted to 80% of water holding capacity (WHC). Soils were incubated at 28°C and CO_2_ emission was estimated every day during 1 week (7 days) using direct injection into a micro GC Analytical Instruments SRA (MTI P200, Microsensor Technology Inc., Fremont, CA, USA).

### Soil Enzymes Assays

FDA hydrolysis was performed as described by Adam and Duncan (2001). Briefly, 2 g soil was placed in a 50 ml conical flask and 15 ml of 60 mM potassium phosphate buffer pH 7.6 were added. Stock solution (0.2 ml 1000 mg FDA ml^-1^) was added to start the reaction. Controls were prepared without the addition of the FDA substrate along with a suitable number of sample replicates. The fluorescein released during the assay was extracted with chloroform/methanol (2:1 v/v) and measured at 490 nm using a spectrophotometer (Spectronic 401, Spectronic Instruments, France). Results were expressed as mg fluorescein released kg^-1^ h^-1^.

Acid phosphatase (EC 3.1.3.2) activity was determined using *p*-nitrophenyl phosphate (5 mM) as substrate. 400 μl of 1 M universal modified buffer at pH 6 and 100 μl of substrate were added to 100 mg of soil and incubated in an orbital shaking incubator (100 rpm) at 37°C for 1 h. The reaction was stopped by adding, respectively, 100 μl of CaCl_2_ 0.5 M and 400 μl of NaOH 0.5 M. The mixture was then centrifuged at 10,000 rpm for 5 min and the *p*-nitrophenol measured using a spectrophotometer set at 400 nm (Tabatabai and Bremner, 1969).

### Statistical Analysis

Analysis of variance was performed to assess the effect of rhizobial inoculation on gum arabic production, soil total microbial biomass, microbial CO_2_ respiration, N content, FDA, and acid phosphatase activities. Principal component analysis was performed to evaluate the correlation between gum arabic production and soil fertility indicators. These tests were performed with the XLSTAT^TM^ software package (version 2009, Addinsoft, Paris, France). Means of these parameters were compared using the Student–Newman–Keuls rang test (*P* < 0.05).

## Results

### Annual Rainfall

Annual rainfall was recorded during the two experimental seasons in order to evaluate the relationship between gum arabic production and rainfall. Rainfall occurred over an extended period of 5 months from June to October, with maximum values in September and August for the 1st and 2nd year, respectively (**Table 2**). Rainfall was higher during the 1st year (495.6 mm) compared to the 2nd year (213.1 mm).

### Rhizobial Inoculation on Soil Microbial Functioning

#### Soil Total Microbial Biomass

Inoculation with selected rhizobial strains brought at the beginning of rainy season around tree trunk had a significant (*P* < 0.05) positive effect on soil total microbial biomass during the two experimental years (**Table 4**). No significant (*P* > 0.05) difference in microbial biomass was observed at the beginning of experiment in soils sampled under uninoculated and trees which be inoculated. However, a significant (*P* < 0.05) effect of inoculation was noted on microbial biomass of soils collected during the rainy season of the 1st year and both dry and rainy seasons of the 2nd year.

**Table 4:** Microbial biomass (μg C g^-1^ dry soil) of soils sampled under uninoculated and inoculated 13-year-old *S. senegal* trees during both dry and rainy seasons of the 2 years.

Treatments	First year		Second year	
	Dry season April	Rainy season August	Dry season April	Rainy season August
Uninoculated trees	^∗^29 ± 2a	39 ± 3a	15 ± 2a	60 ± 6a
Inoculated trees	^∗^31 ± 3a	54 ± 4b	35 ± 5b	80 ± 9b

*Values within a column sharing same letter comparing rhizobial inoculation treatment are not significantly different at *P* < 0.05 (Student–Newman–Keuls test). Each value represents the mean of 12 repetitions (six in each block).*

*^∗^These values were obtained before inoculation.*

#### Soil Microbial CO_2_ Respiration and N Content

Rhizobial inoculation had no significant (*P* > 0.05) effect on CO_2_ produced and nitrogen content (**Table 5**), apart from the rainy season of the 2nd year where CO_2_ produced was significantly lower in inoculated trees.

**Table 5:** Soil microbial CO_2_ respiration (μg C-CO_2_ g^-1^ dry soil/7 days) after 7 days of incubation and nitrogen (μg N g^-1^ dry soil) content of soils sampled under uninoculated and inoculated 13-year-old *S. senegal* trees during dry and rainy season of the 2 years.

Treatments	First year				Second year			
	Dry season April		Rainy season August		Dry season April		Rainy season August	
	C-CO_2_	NH_4_^+^ + NO_3_^-^	C-CO_2_	NH_4_^+^ + NO_3_^-^	C-CO_2_	NH_4_^+^ + NO_3_^-^	C-CO_2_	NH_4_^+^ + NO_3_^-^
Uninoculated trees	^∗^51.9 ± 1.3a	9.1 ± 1.1a	31.1 ± 0.4a	3.7 ± 0.8a	64.7 ± 3.8a	9.7 ± 0.4a	51.4 ± 4.5b	5.1 ± 0.3a
Inoculated trees	^∗^45.4 ± 5.8a	12.8 ± 2.9a	30.6 ± 1.5a	2.4 ± 1.4a	68.2 ± 4.9a	9.1 ± 0.7a	32.2 ± 1.8a	5.8 ± 0.7a

*Values within a column sharing same letter comparing rhizobial inoculation treatment are not significantly different at *P* < 0.05 (Student–Newman–Keuls test). Each value represents the mean of 12 repetitions (six in each block).*

*^∗^These values were obtained before inoculation.*

#### Soil Enzymes Activities

Rhizobial inoculation increased significantly (*P* < 0.05) FDA and acid phosphatase activities of soils sampled during the rainy season of the 2 years (**Table 6**). However, no significant (*P* > 0.05) effect was observed in dry season for both years.

**Table 6:** Fluorescein diacetate (μg fluorescein g^-1^ dry soil h^-1^) and acid phosphatase (μg *p*-nitrophenol g^-1^ dry soil h^-1^) activities of soils sampled under uninoculated and inoculated 13-year-old *S. senegal* trees during dry and rainy season of the 2 years.

Treatments	First year				Second year			
	Dry season April		Rainy season August		Dry season April		Rainy season August	
	FDA	Acid	FDA	Acid	FDA	Acid	FDA	Acid
		phosphatase		phosphatase		phosphatase		phosphatase
Uninoculated trees	^∗^0.91 ± 0.5a	162.48 ± 38.0a	0.49 ± 0.1a	178.60 ± 18.8a	1.27 ± 0.2a	328.58 ± 30.6a	0.91 ± 0.11a	369.14 ± 24.2a
Inoculated trees	^∗^0.86 ± 0.3a	176.01 ± 39.3a	0.84 ± 0.2b	288.49 ± 7.1b	1.38 ± 0.1a	399.70 ± 66.5a	1.27 ± 0.16b	457.7 ± 35.8b

*Values within a column sharing same letter comparing rhizobial inoculation treatment are not significantly different at *P* < 0.05 (Student–Newman–Keuls test). Each value represents the mean of 12 repetitions (six in each block).*

*^∗^These values were obtained before inoculation.*

### Effect of Rhizobial Inoculation on Gum Arabic Production by 13-Year-old *S. senegal* Trees

**FIGURE 1:**
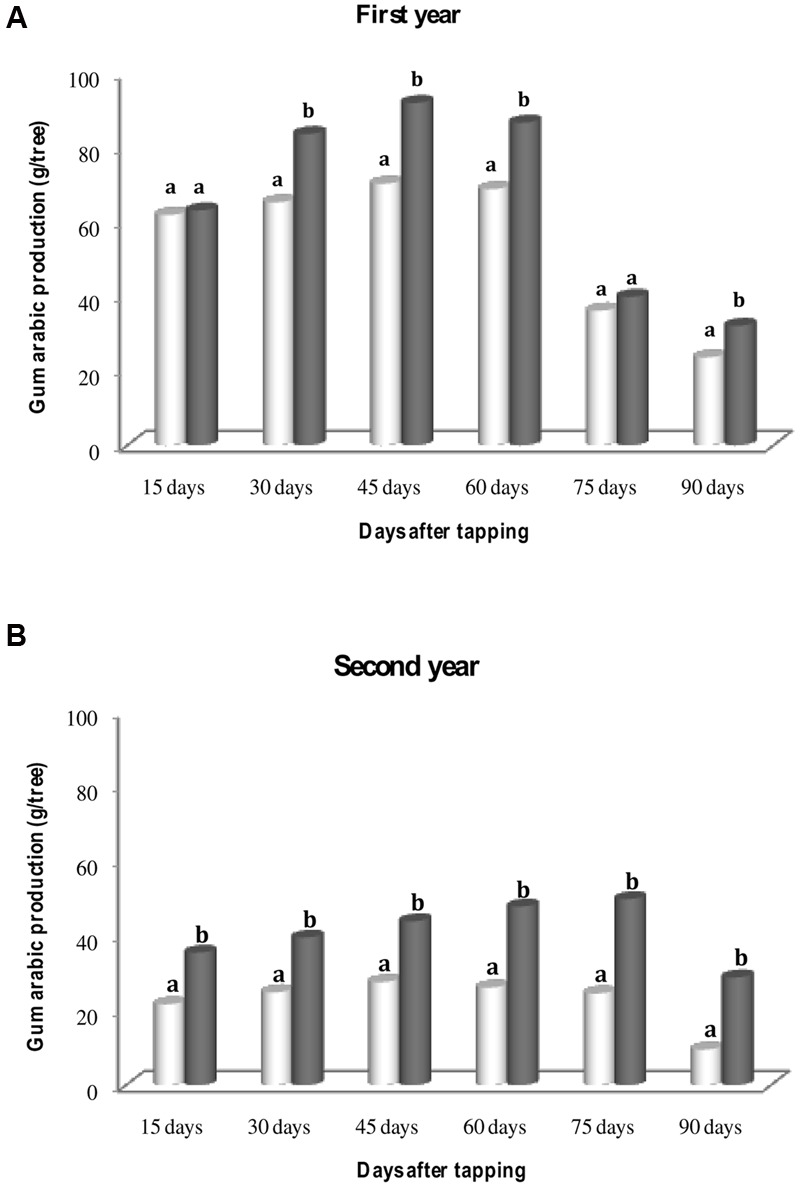
**Gum arabic produced every 15 days for 3 months by uninoculated and inoculated 13-year-old *S. senegal* trees during 1st year **(A)** and 2nd year **(B)**.** For each date of harvest, bars with same letter comparing rhizobial inoculation effect are not significantly different at *P* < 0.05 (Newman–Keuls test).

**FIGURE 2:**
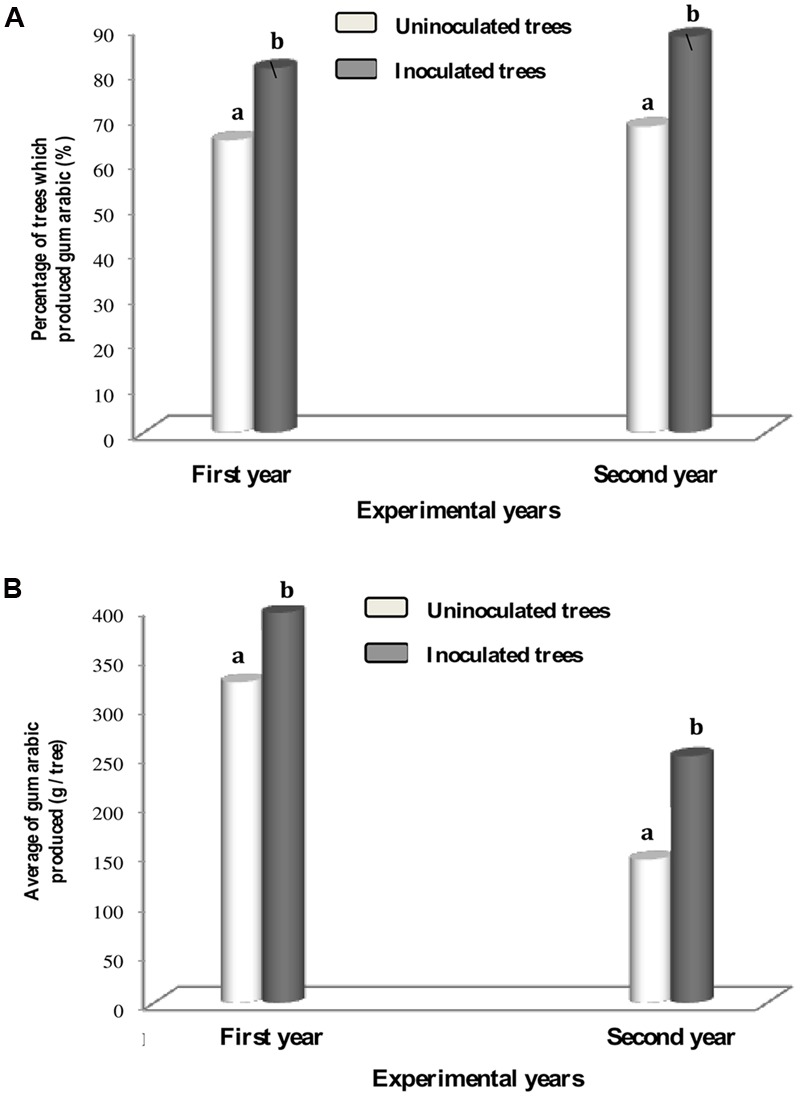
**Percentage of gum arabic producing trees **(A)** and the yield of gum arabic produced **(B)** by uninoculated and inoculated 13-year-old *S. senegal* trees in 1st and 2nd year.** For each year, bars with same letter comparing rhizobial inoculation effect are not significantly different at *P* < 0.05 (Newman–Keuls test).

**Figure 1** showed that gum production was maximal 45 days after tapping for the 1st year for uninoculated and inoculated trees (**Figure 1A**). During the second experimental year, the maximum of gum arabic was always recorded 45 in uninoculated and 75 days after tapping in inoculated trees (**Figure 1B**). Percentage of uninoculated and inoculated trees producing gum arabic and the average of gum arabic production per tree at the end of first and second harvest seasons are represented in **Figures 2A,B**. For each experimental year, percentage of inoculated trees that produced gum arabic was significantly (*P* < 0.05) higher than that of uninoculated trees (**Figure 2A**). Percentages of gum arabic production in first and second experimental years were 81 and 88%, respectively in inoculated trees whereas they were 65 and 68% for uninoculated trees. Results obtained at the end of each experimental season showed that inoculation significantly increased (*P* < 0.05) gum arabic production per tree (**Figure 2B**). Inoculated trees produced an average of 396.11 g and 250.39 g per tree during the 1st and 2nd years, respectively. By contrast, the average amount of gum arabic produced by uninoculated trees was 325.79 g per tree in the 1st year and 145.45 g per tree in the 2nd year. However, this positive effect of rhizobial inoculation was more pronounced during the 2nd year. For both treatments (uninoculated and inoculated), gum arabic production per tree was higher in the 1st year than in 2nd year (**Figure 2B**). However, this decrease in gum arabic production between years was more pronounced in uninoculated trees compared to inoculated trees (55.35 vs. 36.79%).

### Relationships between Assessed Parameters

**FIGURE 3:**
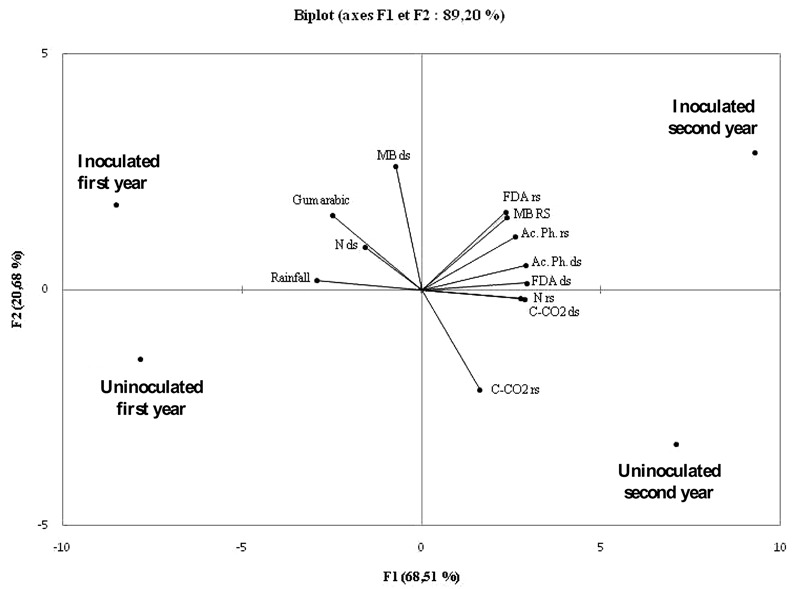
**Principal component analysis showing the correlation between gum arabic production and rainfall, soil total microbial biomass, soil microbial CO_2_ respiration, N content, FDA, and acid phosphatase activities.** rs, rainy season; ds, dry season; MB, microbial biomass; N, mineral nitrogen; Ac. Ph., acid phosphatase.

The matrix of correlation indicated that the gum arabic production was positively correlated with rainfall (*R* = 0.88), soil total microbial biomass (*R* = 0.86) and mineral N content in dry season (*R* = 0.62). Significant (*P* < 0.05) positive correlation was also observed between FDA and acid phosphatase activities (*R* = 0.98), which both were positively correlated with soil microbial biomass (*R* > 0.95). A significant (*P* < 0.05) negative correlation was observed between rainfall and FDA (*R* = -0.98), acid phosphatase (*R* = -0.97) activities, CO_2_ produced (*R* = -0.96) and nitrogen content (*R* = -0.92). **Figure 3** showed that the first two axes (F1 and F2) explained 89.20% of data variability, suggesting that rhizobial inoculation influenced the assess parameters. During the 1st year, the parameters were negatively correlated to F1 and positively correlated to this same axis during the 2nd year. Excepted microbial biomass in dry season and CO_2_ produced in rainy season, which were correlated to F2, all soil parameters were positively correlated to F1. However, gum arabic and rainfall were negatively correlated to F1. Globally, soil microbial functioning parameters were increased by inoculation during the 2nd year. However, gum arabic production was higher during the first and was positive correlated to rainfall.

## Discussion

Our results showed that rhizobial inoculation increased soil microbial functioning in natural conditions as shown in greenhouse conditions (Bakhoum et al., 2012). Rhizobial inoculation increased significantly soil total microbial biomass. Similar results were obtained by Marion et al. (2004); Sun et al. (2009), Teng et al. (2011), and Bakhoum et al. (2012). However, our results contrast with those reported by Johnson et al. (2005) which indicated that rhizobial inoculation did not significantly increase total soil microbial biomass. This may be due partly to the experimental conditions. Indeed, their study was conducted under controlled laboratory conditions with polycyclic aromatic hydrocarbons (PAH) contaminated soil and the PAH could drastically influence the bacterial proliferation in soil. Moreover, they used 180 days old ryegrass/clover in contrast to our study which was carried out under natural condition with rhizospheric soils of 13 years old *S. senegal* mature trees. In Johnson et al. (2005), quantity and/or quality of root exudates may not be sufficient to stimulate the proliferation of soil microbial communities to enhance total soil microbial biomass. Total microbial biomass (bacteria and fungi) is a measure of the mass of the living component of soil organic matter. The positive effect of rhizobial inoculation on total soil microbial biomass can be due to a proliferation of Proteobacteria, Firmicutes, and Actinobacteria (Trabelsi et al., 2012). Indeed, Trabelsi et al. (2011) showed that the mono- and dual inoculation with *Rhizobium gallicum* strain 8a3 and *Ensifer meliloti* strain 4H41 induced the proliferation of bacterial communities that had been frequently reported as plant growth-promoting microorganisms, like, *Bacillus, Azospirillum, Mesorhizobium, Pseudomonas*,…. In addition, previous studies have indicated that rhizobia can increase exudation from host plant roots and secondary plant metabolites such as luteolin (Peters et al., 1986; Phillips and Streit, 1996). Thus, increased amounts of exudates may in turn support the growth of microbial communities in particular degraders (Teng et al., 2011).

By contrast, no significant effect of rhizobial inoculation was observed on soil CO_2_ produced after 7 days and N content apart from for CO_2_ produced in dry season of the 2nd year. The lack of rhizobial inoculation effect on C and N mineralization could be attributed to the duration of our experiment. Indeed, Sall et al. (2007) showed that the type of residue did modify C mineralization after 134 days of incubation. The negative effect of rhizobial inoculation on mineral C content during the rainy season of the 2nd year could be attributed to an immobilization of inorganic C by soil microorganisms (Sall et al., 2007; Fall et al., 2012). FDA hydrolysis has been widely used as accurate, sensitive, and simple method for measuring total microbial activity in soil (Schnürer and Rosswall, 1982; Nannipieri et al., 2003). Rhizobial inoculation increased significantly microbial total activities and acid phosphatase activities of soils sampled during rainy season, in agreement with several other studies which have demonstrated that root nodulating-bacteria produce acid phosphatase (Chen et al., 2001; Pongsilp et al., 2010). Rhizobial inoculation could improve microbial biomass or diversity through favorable microenvironment caused by plant root exudates and then, enhances the total microbial activity in the soil. However, no significant effect of rhizobial inoculation on these parameters was observed during the dry season, which could be attributed to low soil moisture. It has been demonstrated that soil water content is one of the most important factors controlling soil enzymes activities (Ye et al., 2009; Jang and Kang, 2010). The negative correlation between rainfall and soil microbial functioning parameters shown in our results can be explained by high soil moisture (88–94% of WHC), which inhibits soil microbial activities. Indeed, soil microbial activities are highest when soil moisture is between 60 and 80% of WHC.

Rhizobial inoculation of 13-year-old *S. senegal* trees with selected rhizobial strains increased significantly percentage of gum arabic producing trees and amount of gum arabic produced by each tree. Such positive effects on gum arabic production might be attributed to mineral nutrition improvement of trees, mainly through rhizobial symbiosis since uninoculated and inoculated trees had the same age, similar morphometric characteristics (collar diameter and height) and also were grown on the same soil. However, assessment of nitrogen fixation in both uninoculated and inoculated trees might clarify the relation between nitrogen and gum arabic production. A similar study taking into account assessment of nitrogen fixed by inoculated and uninoculated trees must be done in several contrasting sites to confirm our findings. Furthermore, this positive effect of rhizobial inoculation on gum arabic production may also be due in part to its positive effect on soil fertility as shown by result on soil microbial soil functioning. Similar results were obtained by Faye et al. (2006) with the same rhizobial strains and 10-year–old *S. senegal* trees in Rotto-Senegal.

Although a decreasing amount of gum arabic was observed during the 2nd year, the overall gum arabic production was positively correlated to rainfall, which is in concordance with results previously reported by Bateson et al. (1988) and Ballal et al. (2005). One possible explanation of these observations is that the decrease of rainfall during the 2nd year (213.1 vs. 495.6 mm in the 1st year) had negatively impacted gum arabic production. During the first season, rainfall received was probably more favorable for gum arabic production compared to 2nd year. In agreement with this assumption, Dommergues et al. (1999) demonstrated that rainfall needed by *S. senegal* to grow properly and produce more gum arabic during next harvest season is about 450 mm.

## Conclusion

Our results showed that inoculation of mature *S. senegal* trees in natural conditions with selected rhizobial strains increased significantly gum arabic production, soil total microbial biomass, total microbial activities and acid phosphatase. By contrast, rhizobial inoculation did not affect soil microbial CO_2_ respiration and N content. Gum arabic production was positively correlated to rainfall, soil total microbial biomass and N in dry season. Strong positive correlation was found between soil enzyme activities and soil microbial biomass. These important findings in natural conditions deserve to be conducted in several contrasting sites.

## Author Contributions

All authors listed, have made substantial, direct and intellectual contribution to the work, and approved it for publication.

## Conflict of Interest Statement

The authors declare that the research was conducted in the absence of any commercial or financial relationships that could be construed as a potential conflict of interest.

The reviewer VV and handling Editor declared their shared affiliation, and the handling Editor states that the process nevertheless met the standards of a fair and objective review.
